# Taxonomic studies on the Chara section Hartmania in Poland based on morphological and molecular data

**DOI:** 10.3897/phytokeys.135.36714

**Published:** 2019-12-05

**Authors:** Jacek Urbaniak, Paweł Kwiatkowski

**Affiliations:** 1 Department of Botany and Plant Ecology, Wrocław University of Environmental and Life Sciences, Poland Wrocław University of Environmental and Life Sciences Wroclaw Poland; 2 Institute of Biology, Biotechnology and Environmental Protection, University of Silesia in Katowice, Poland University of Silesia in Katowice Katowice Poland

**Keywords:** Chara, morphological characters, species identification, taxonomy, Poland

## Abstract

Charophytes are aquatic green macroalgae, which inhabit fresh and brackish water ecosystems. In this study, four species belonging to the genus *Chara* were examined to determine their taxonomic status. Morphological characteristics of the plant bodies as well as plastid *psa*B barcoding genes were applied to test the relations among *Chara* species. Plants were initially classified using morphological features into four species: *C. baltica*, *C. hispida*, *C. polyacantha* and *C. rudis*, and twelve quantitative characters were used in a principal component analysis and discriminant analysis to determine groupings among the species and to determine the morphological features that best separated the groups. In the component analysis and discriminant analysis, results showed that only *C. polyacantha* and partly *C. baltica* formed separate groups. The other species *C. hispida* and *C. rudis* were only partially distinguishable. All species from one molecular group, and no differentiation in the *psa*B variability between them has been found.

## Introduction

Macroscopic algae from the genus *Chara* L. can be commonly found in various water bodies, such as shallow lakes, artificial ponds, slowly running waters or drainage canals. The taxonomy of the genus *Chara*, as well as the other representatives of the Characeae family is not easy, mostly due to the overlapping of morphological features of individual specimens belonging to different species (Sakayama et al. 2002; Nylander et al. 2004; Sakayama et al. 2009; Urbaniak 2010, 2011a, 2011b; Urbaniak and Combik 2013; Schubert 2014). The variability among specimens is probably also due to genetic and ecological (environmental, site-specific) conditions (e.g. water quality, light availability) and resulting phenotypic plasticity or developmental differences (Meiers et al. 1999; Mannschreck 2003).

Taxonomic studies based on charophyte morphology started at the end of the 19^th^ century, and during this initial phase, many people tried to characterize the degree of morphological variation in charophytes and find traits to circumscribe distinct species. Traditionally, in the genus *Chara*, a narrow species concept has been used resulting in about 45 European species (Braun and Nordstedt 1882; Corillion 1957; Krause 1997). Because of overlapping morphological variations in many traits, some workers have used a wider species concept and interpreted the genus to be subdivided into fewer and more polymorphic species (Wood and Imahori 1965) thus discriminating only 18 species (worldwide) including a number of varieties and forms. These differences between understanding and interpreting the species result from a lack of objective methods to determine which characters actually serve to delimit species within the genus. This problematic classification is typical not only for the genus *Chara* (Mannschreck 2003; O’Reilly et al. 2007; Urbaniak and Combik 2013) but also for the genus *Nitella* C.Agardh (Sakayama et al. 2002). Because certain intermediate forms exist between *C. baltica*, *C. hispida*, *C. polyacantha* and *C. rudis*, authors treat them in different ways: they either consider them to be separate species (Krause 1997; Urbaniak and Gąbka 2014) or as varieties or forms of *C. hispida* (Wood 1962; Wood and Imahori 1965) (Table 2).

**Table 1.** Specimens studied with GenBank accession numbers and collection sequences used in study.

Species	*p*saB GenBank accession number/locality	Geographical coordinates
*C. baltica* Bruz.	KX791851/ Puck, Poland	54°42'14.09"N, 18°27'40.70"E
	KX791852/ Swarzewo, Poland	54°45'25.19"N, 18°24'33.91"E
	KX791853/ Rewa, Poland	54°38'11.17"N, 18°30'37.50"E
*C. hispida* L.	KX791854/ Lake Czarne, Poland	54°00'57.75"N, 22°59'40.22"E
	KX791855/ Lake Mikaszewo, Poland	53°53'15.96"N, 23°21'22.84"E
	KX791856/ Lake Białe, Poland	53°52'03.95"N, 23°02'16.17"E
	KX791857/ Lake Czajcze, Poland	54°06'56.00"N, 22°28'19.44"E
	KX791858/ Lake Wielkie, Poland	53°20'43.74"N, 22°55'58.36"E
	KX791859/ Lake Wigry, Poland	54°00'34.76"N, 23°02'16.89"E
	KX791860/ Lake Mamry, Poland	54°06'24.87"N, 21°46'13.13"E
	KX791861/ Lake Pobłądzie, Poland	54°18'24.30"N, 22°45'25.25"E
	KX791862/ Lake Muliste, Poland	53°54'11.23"N, 23°16'15.58"E
	KX791863/ Lake Staw, Poland	54°01'14.44"N, 22°59'26.91"E
	KX791864/ Lake Jeziorak, Poland	53°43'06.51"N, 19°36'02.98"E
	KX791865/ Lake Śniardwy, Poland	53°47'25.95"N, 21°44'14.71"E
*C. polyacantha* A. Br.	216/KX791866/ Lake Jasne, Poland	54°07'56.82"N, 22°58'41.09"E
	217/KX791867/ Lake Bilskie, Poland	54°05'03.47"N, 23°05'31.62"E
	218/KX791868/ Lake Wigry, Poland	54°01'48.15"N, 28°08'30.31"E
	219/KX791869/ Lake Kockie, Poland	53°59'40.25"N, 20°51'25.79"E
*C. rudis* Leonh.	220/KX791870/ Lake Staw Wielki, Poland	53°57'01.46"N, 23°08'42.92"E
	221/KX791871/ Lake Oleckie, Poland	54°03'23.86"N, 22°30'20.63"E
	222/KX791872/ Lake Małe, Poland	54°03'24.09"N, 22°42'09.85"E
	223/KX791873/ Lake Korzęckie, Poland	54°13'29.08"N, 22°34'05.47"E
*C. vulgaris* L.	DQ229107/Poland	–
*N. axiliformis*	AB191785/Japan	–
*N. pseudoflabellata*	AB191766/Japan	–

**Table 2.** Classification of selected species from the genus *Chara* (Charophyta).

Krause (1997)	Wood and Imahori (1965)	Becker et al. (2016)
*Chara baltica* Bruz.	Chara hispida var. baltica f. baltica	*Chara baltica* (Hartman) Bruz.
	Chara hispida var. baltica f. liljebladi	
*Chara hispida* L.	Chara hispida var. major f. major	*Chara hispida* L.
	Chara hispida var. hispida f. hispida	
*Chara polyacantha* A. Braun	Chara hispida var. hispida f. polyacantha	*Chara aculeolata* Kütz.
*Chara rudis* (A. Braun) Leonh.	Chara hispida var. major f. rudis	*Chara subspinosa* Rupr.

Unfortunately, previous studies of oospore morphology, oospore wall ornamentation (scanning electron microscopy, SEM) and molecular fingerprinting data did not give satisfactory results in delimitating Chara species from the section Hartmania (Urbaniak and Combik 2013). This could indicate, that *i*) the choice of the method used was not the best solution or *ii*) it showed a very close phylogenetic relationship among *C. baltica*, *C. hispida*, *C. polyacantha* and *C. rudis* and all these taxa should be treated as varieties or forms of *C. hispida* according to the monomorphic species concept (Wood and Imahori 1965). SEM studies of the oospore wall ornamentation and dimensions have also been used for species delimitation in the genus *Chara* to suggest that both methods can be helpful in taxonomic decisions regarding species (John et al. 1990; Urbaniak 2011a, 2011b; Urbaniak and Blazencic 2012). Sakayama et al. (2002) showed that the combination of different types of data (SEM, oospore morphology and molecular data) can be more informative than when considered separately and can be used for taxonomic distinction, especially in closely related species of the genus *Nitella* Agardh.

In addition, the DNA barcoding method has been proposed as an alternative method for identifying taxonomic relationships in species of the Characeae family. This method can be used successfully to facilitate biodiversity and taxonomic studies of various plants (Kress et al. 2005). Sakayama et al. (2002) applied different barcoding genes of *mat*K, *rbc*L or *psa*B genes to test whether the distribution of haplotypes among individuals is consistent with species boundaries as they are currently understood. The choice of *rbc*L+*mat*K as a barcode was probably based on the good recovery of the *rbc*L region and high discriminatory power of the *mat*K region, which is one of the most rapidly evolving coding sections of the plastid genome (Hollingsworth et al. 2011). However, Hollingsworth et al. (2009) as well as Shaw et al. (2007) point out that the plastid barcode gene *psa*B can be used that serves good delimitation. The use of *psa*B gene has been tested previously with good results by Sakayama et al. (2005) in a taxonomic re-examination of the genus *Nitella*.

The presented study focuses on four of the most problematic freshwater species (two diplostichous aulacanthous species, *C. hispida* and *C. rudis*, one diplostichous thylacanthous species: *C. polyacantha* and *C. baltica* as representatives of brackish water species (a diplostichous thylacanthous species, in transition to slightly isostichous). All of them belong to the section Hartmania. We applied the plastid *psa*B gene as well as morphological observation to test whether the distribution of haplotypes among species is in agreement with the species delimitation.

## Methods

### Collection of plants and PCR analysis

The plants were collected manually or using a hook directly from the field. We collected mature specimens and determined according to the Krause (1997), Urbaniak and Gąbka (2014), Becker et al. (2016) and van de Weyer (2016) determination keys.

We have used the charophyte names following Krause (1997) and Urbaniak and Gąbka (2014): *C. baltica*, *C. hispida*, *C. polyacantha* and *C. rudis*. In case of *C. rudis* a name on species rank that has priority was established recently: *Chara subspinosa* Rupr. An earlier name for the widely used name for *C. polyacantha* is *C. aculeolata* Kütz. (Becker et al. 2016), however, in this case its taxonomic position is not clear. The synonymy is presented in Table 2. In the case of molecular analysis, after collection, the material was placed in glass jars and transported to the laboratory and cultured in laboratory conditions (at room temperature, with light from a north-facing window) in jars filled with tap water. To reduce the influence of contaminating DNA from epiphytes, large filamentous green algae were removed from young plant shoots before DNA extraction by dissection under a stereomicroscope. Only newly grown tissue was used for molecular analysis.

### Morphological observations

**Figure 1.**
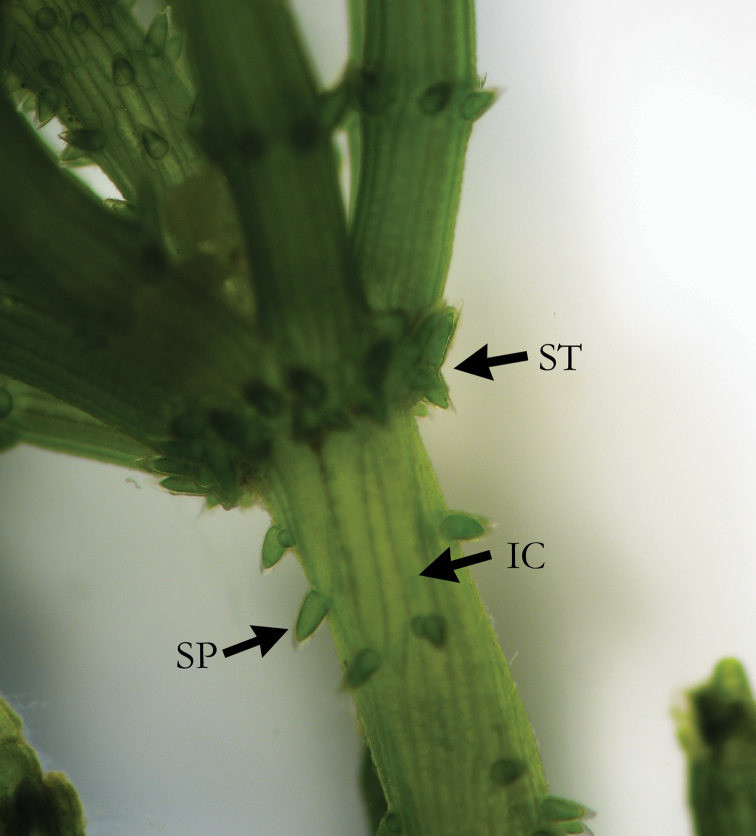
*C. baltica* with short irregular stipulodes (ST), spine cells (SP) shorter than axis diameter, and irregular cortex (IC).

**Figure 2.**
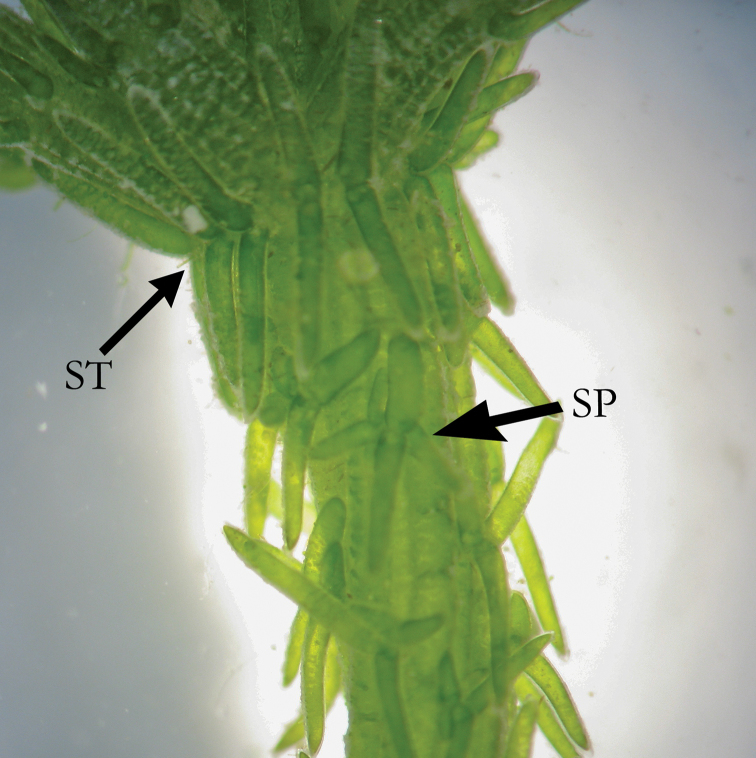
*C. hispida* with stipulodes (ST) and spine cells (SP) in similar length, as long as axis diameter.

**Figure 3.**
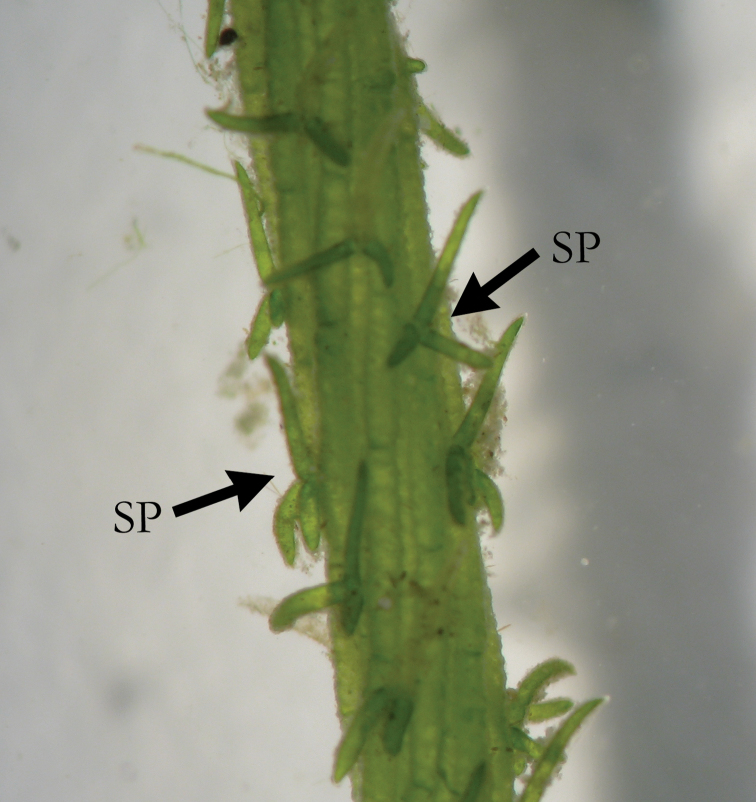
*C. hispida* with spine cells (SP) in bunches.

**Figure 4.**
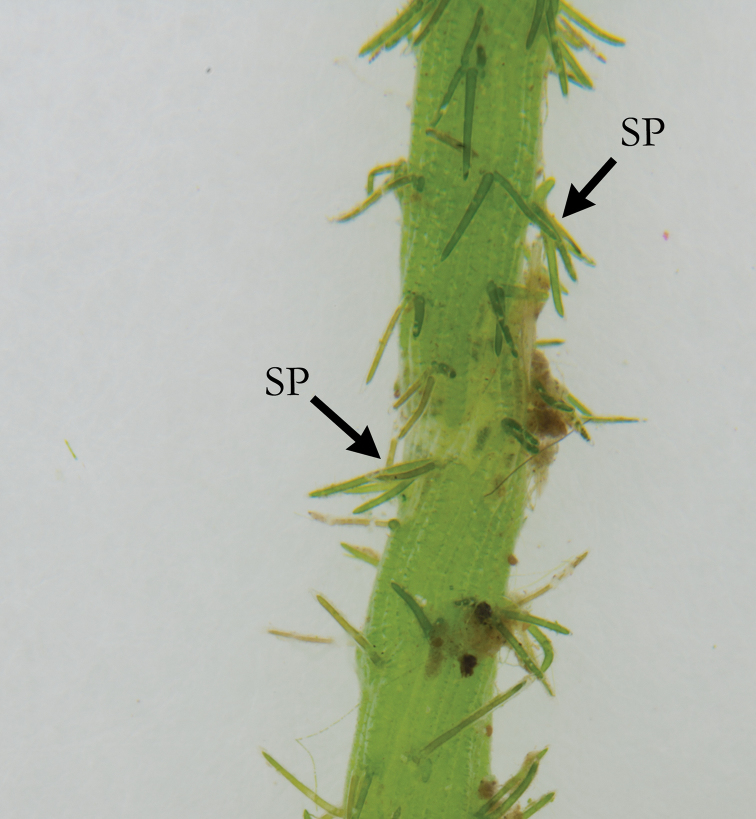
*C. polyacantha* with long spine cells (SP), as long as axis diameter.

**Figure 5.**
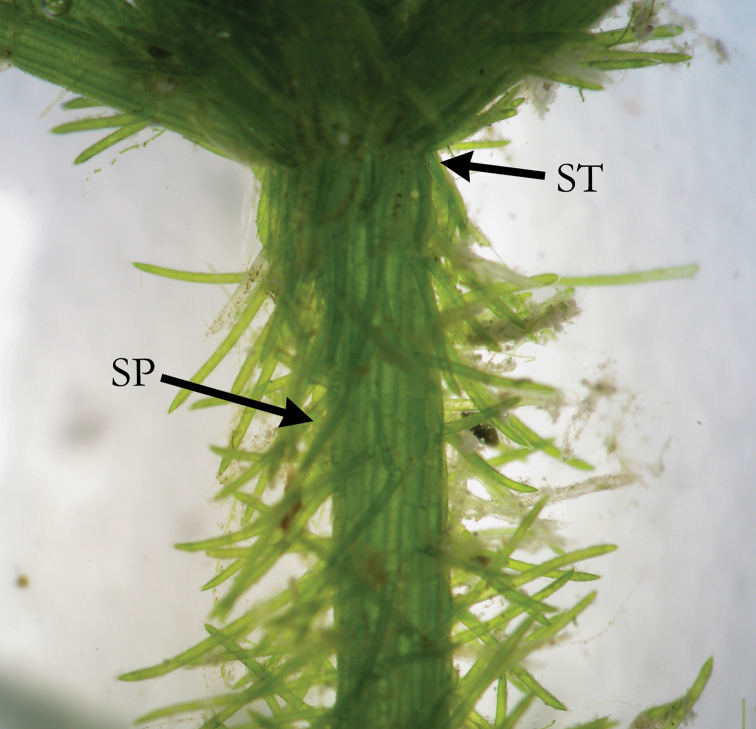
*C. polyacantha* with extremely long spine cells (SP) and stipulodes (ST) exceeding axis diameter.

**Figure 6.**
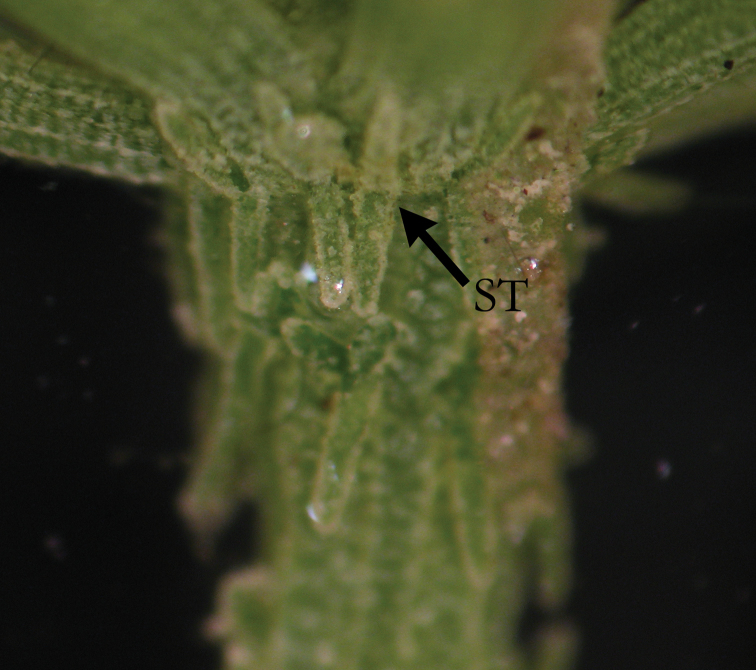
*C. rudis* with short, regular stipulodes (ST).

**Figure 7.**
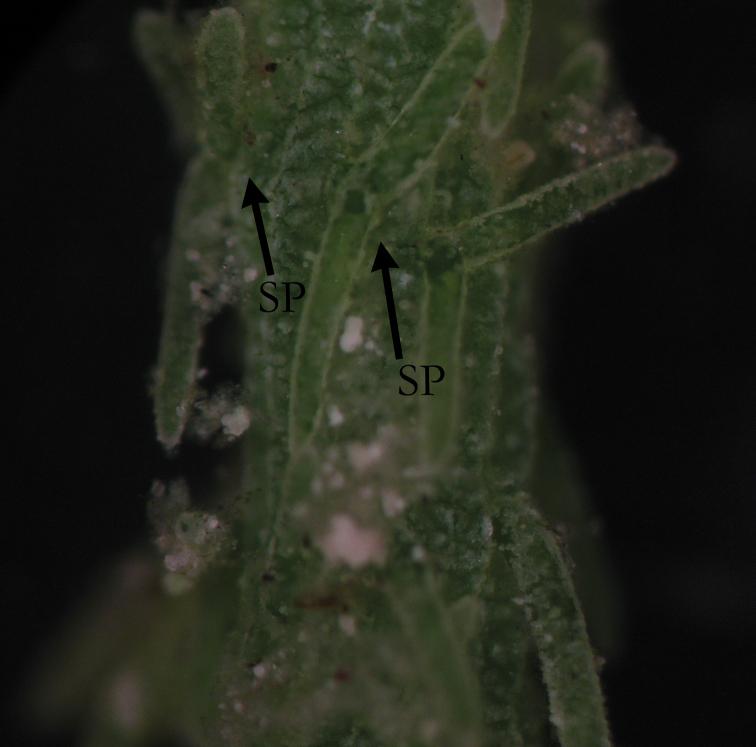
*C. rudis* with opposite spine cells (SP) on the axis.

**Figure 8.**
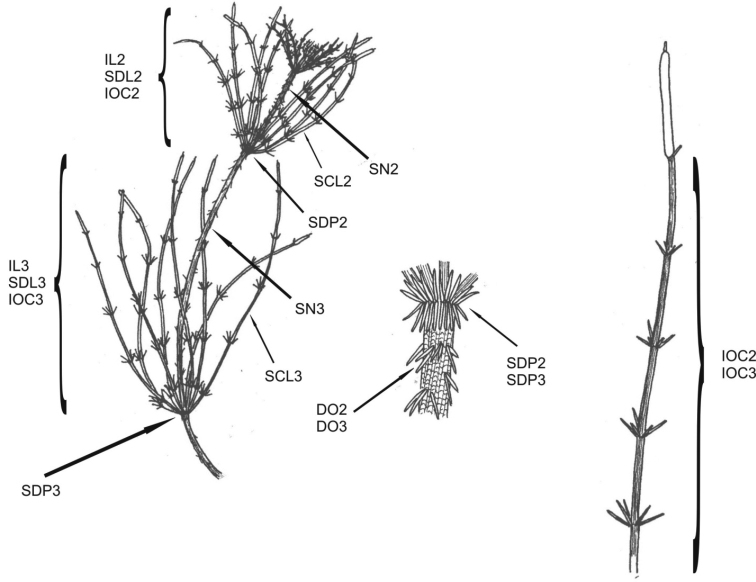
Axis, internode and node complex and branchlet characteristics measured in this study. Abbreviations of the morphological features are given in Table 4. Figure after Bruinsma et al. (1988), modified.

In the case of morphological observations, after collection, plants were dried and analysed in a laboratory using a stereomicroscope SMZ 800 (Nikon, Tokyo, Japan). The morphological characteristics of the investigated species were described (Table 3) with some examples of studied species with important discriminatory analysis shown in Figs 1–7. The characters used for performing the principal component analysis (PCA) and discriminant analysis (DCA) are shown in Fig. 8, coded and analysed using PCA and DCA discriminatory techniques using Statistica 12.1 software (StatSoft 2010). The morphological features (quantitative characters) used in the analysis for species in Chara section Hartmania are described in Table 4. No fewer than 30 specimens have been measured in this instance, except for the population *C. polyacantha* (Lake Wigry, Bilskie, Kockie), where only about 23 specimens have been analysed.

**Table 3.** Comparisons of morphological features of studied species.

Character / Feature	*C. baltica*	*C. hispida*	*C. polyacantha*	*C. rudis*
Plant axis	robust, slender	robust, thick	erect, robust	robust, thick
Plant size	medium size, 6–27 cm high, 1–2 mm in diameter	medium large to large species, up to 18–70 cm high, 4–5 in diameter	medium large to large, 30–75 cm high, up to 5 mm in diameter	medium large to large plants, 23–65 cm high, up to 4–6 mm in diameter
Color	light to dark green	green to greyish green	green to dark green	green to greyish green
Incrustation	unincrusted	moderately to heavily incrusted	moderately incrusted	moderately to heavily incrusted
Internodes	longer or as long as branches	longer than branches	similar length or longer (up to 2 times) than branches	up to 2 times longer than branches
Branchlet	up to 8 branches in a whorl, stout to slender with 5–8 segments	7–10 branches in a whorl, straight and rigid, with 5–9 segments	8–10 branches in a whorl with 6–9 segments	7–10 branches in a whorl, with 6–7 segments
Cortification	diplostichous and slightly thylacanthous	diplostichous, aulacanthous, often isostichous on older internodes	diplostichous and tylacanthous occasionally irregular	diplostichous, strongly heterostichous and aulacanthous
Spine cells	shorter than axis diameter, solitary or in pairs	solitary or in fascicles as long as the axis diameter	in bunches, as long or longer than the axis diameter	sparse in pairs, similar in length as plant axis
Stipulodes	stipulodes in two rows similar in length to spine cells	stipulodes in two rows, uppers are similar to lowers	stipulodes in two rows, as long as axis diameter	stipulodes are in two rows, uppers similar to lowers
Reproduction	monoecious	monoecious	monoecious	monoecious
Oogonia	540–1165 μm long, 515–650 μm wide	415–1200 μm long, 520–770 μm wide	625–1140 μm long, 450–615 μm	890–1210 μm long, 415–750 μm wide
Antheridia	420–630 μmin diameter	490–730 μm in diameter	375–530 μm in diameter	370–480 μm in diameter
Oospores	black, 465–925 μm long and 335–645 μm wide	reddish brown to dark brown, 545–810 μm long, 390–760 μm wide	brown, dark brown or black, 485–900 μm long, 270–585 μm wide	brown to dark brown–almost black, 620–925 μm long, 395–835 μm wide

**Table 4.** Morphological features used in the analysis of features in four species in Chara section Hartmania. See Figure 1 for a diagrammatic explanation. Qualitative characters are signed with “[n]” and quantitative with “[cm]”.

Feature (see Figure 1)	Abbreviation
Number of branches in second branchlet whorl [n]	IL2
Mean length of branches in second branchlet whorl [cm]	SDL2
Mean number of corticated internodes on branchlets in the second whorl [n]	IOC2
Mean length of stipulodes at the second node [cm]	SDP2
Diameter of the internode above second branchlet whorl [cm]	SN2
Length of spine cells above second branchlet whorl [cm]	DO2
Number of branchlets in third branchlet whorl [n]	IL3
Mean length of branchlets in third branchlet whorl [cm]	SDL3
Mean number of corticated internodes on branchlets in third whorl [n]	IOC3
Mean length of stipulodes at the third node [cm]	SDP3
Diameter of the internode above third branchlet whorl [cm]	SN3
Length of spine cells above third branchlet whorl [cm]	DO3

### Molecular analysis

In addition to the morphological observations, a molecular technique, sequencing of the plastid *psa*B gene, has been conducted. Total genomic DNA was isolated from fresh tissue using liquid nitrogen and a DNeasy Plant Mini Kit (Qiagen, Hilden, Germany) according to the manufacturer’s protocol. Cells were disrupted using the Mixer Mill MM400 (Retsch, Haan, Germany). The quality and quantity of the DNA was determined on 1% TBE–agarose gel. The PCR amplification and sequencing of the *psa*B gene was accomplished using the primers described by Sakayama (2008). Analyses were performed in a GeneAmp 9700 Thermal Cycler (Applied Carlsbad, CA, USA). Each 20 μl reaction contained water, 10 mM each of dATP, dCTP, dGTP, and dTTP; 0.5 μM of each primer, 10.0 μl reaction buffer, 0.2 μl DreamTaq DNA Polymerase (Thermo Scientific, Waltham, MA, USA) and 1.0 μL of total genomic DNA. The PCR cycle consisted of an initial denaturation at 95 °C for 6 min., followed by 33 cycles at 95 °C for 45 sec., followed by testing the adequate annealing temperature for 45 sec., and elongation 72 °C for 1 min, with a final extension of 10 min at 72 °C. The PCR products were examined for correct length, yield and purity under UV light on 1% agarose gels, stained with SimplySafe. PCR products were purified prior to sequencing reactions, using the Exo–BAP Mix (Eurx, Gdańsk, Poland), and sequenced using the amplification primers. All molecular analyses were performed at the Department of Botany and Plant Ecology, Wrocław University of Environmental and Life Sciences.

### Phylogenetic analysis

Prior to the phylogenetic analyses, the *psa*B DNA sequences were aligned using CLUSTAL W (Thompson et al. 1994). A tree was constructed using PHYML 3.0 by the maximum likelihood (ML) method (Guindon and Gascuel 2003). Prior to analysis, the KAKUSAN 4 (Tanabe 2011) was used to identify the sequence evolution model that fit the dataset using Akaike’s Information Criterion (AIC). The bootstrap proportions (BP) (Felsenstein 1985) used for ML analyses and selected with the GTR + G model selected by KAKUSAN 4 were calculated based on 100 replicates of heuristic searches. The BI analyses were performed using MRBAYES 3.1.2. (Ronquist and Huelsenbeck 2003). The Bayesian inference (BI), were also constructed and compared the topologies of the obtained trees to establish and validate the phylogenetic position of the studied species. The substitution models used for each codon position of the *psa*B gene in the BI analyses were GTR + I (1^st^ codon position), GTR + I + G (2^nd^ codon position), and GTR + G (3^rd^ codon position), which were estimated based on AIC and selected by MRMODELTEST 2.3 (Nylander et al. 2004) implemented in PAUP* 4.0b10 (Swofford 2002). The parameters of the substitution models for each codon position were unlinked. The Markov chain Monte Carlo iteration process was stopped at 1,000,000 generations, and the first 25% of generations were discarded as burn-in, whereas the remaining trees were used to calculate a 50% majority–rule tree and to determine the posterior probabilities (PP) of individual branches.

## Results

The specimens examined in the present study are described in detail in Table 3. In general, all plants were robust and thick, medium to large with plant axis up to 4–6 mm in diameter, except for *C. baltica*, which has a thinner main axis. All plants differed in colour and level of incrustation that determines colour a little. Differences were also noted in the size of internodes and number of branches. All specimens were diplostichous, but sometimes thylacanthous or with occasionally aulacanthous cortex (*C. hispida*, *C. rudis*). The studied plants were monoecious with stipulodes in two rows with spine cells shorter than the axis diameter (*C. baltica*) or with spine cells longer or as long as the axis (*C. hispida*, *C. polyacantha*, *C. rudis*). The details of gametangia (oospores, oogonia and antheridia) are in Table 3. All investigated taxa grow in similar water places except *C. baltica*, which is a truly brackish water species and can be found only in the Baltic Sea. The others are cosmopolitan species, found in different aquatic habitats such as lakes, ponds, pools and petland exploitation pools, with a wide ecological range, growing in both mesotrophic and eutrophic water.

**Figure 9.**
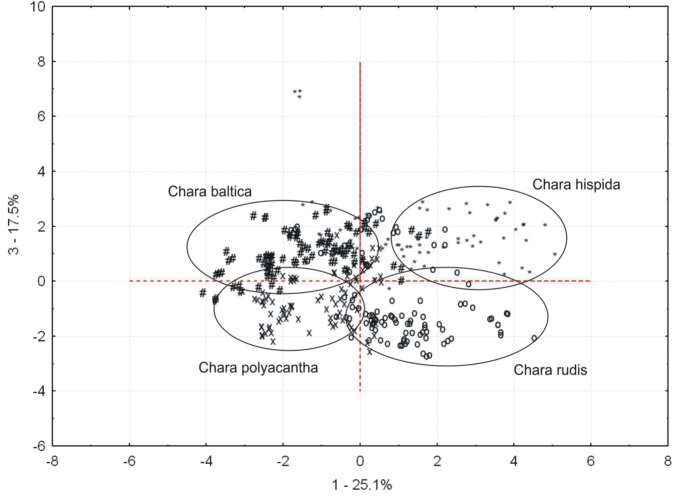
Principal Components Analysis ordination of the species from section Hartmania.

The first three components in the PCA explained 25.1%, 21.3% and 17.5% of the total morphological variation. Four groups that correspond to the four species can be distinguished along the first and third axes (Fig. 9). Specimens that key out to *Chara rudis* and *C. polyacantha* were separated from specimens that key out to the other two species along the first axis. Specimens that key out to *Chara polyacantha* and *C. Baltica* were separated from the other specimens along the third axis. Some specimens assigned to species using the conventional key characters were incorrectly grouped in the PCA, and this occurred for all species. The first component that separated the specimen groups (PC1, 25.1%), was made up largely of mean branchlet length in the second and third branchlet whorls, the diameter of the internode above the second branchlet whorl and length of spine cells above the third branchlet whorl. This component resulted in positive values for specimens that key out to *C. hispida* and *C. rudis* in contrast to specimens that keyed out to *C. baltica* and *C. polyacantha*, which had negative values. The third component (PC3, 17.5%) was made up of differences in the mean number of branchlets in the second and third branchlet whorls and the diameter of the internode above the third branchlet whorl. Specimens that keyed out to *C. polyacantha* and *C. rudis* had negative values in this component, which allowed them to be distinguished from specimens that keyed out to *C. baltica* and *C. hispida*, which had positive values.

**Figure 10.**
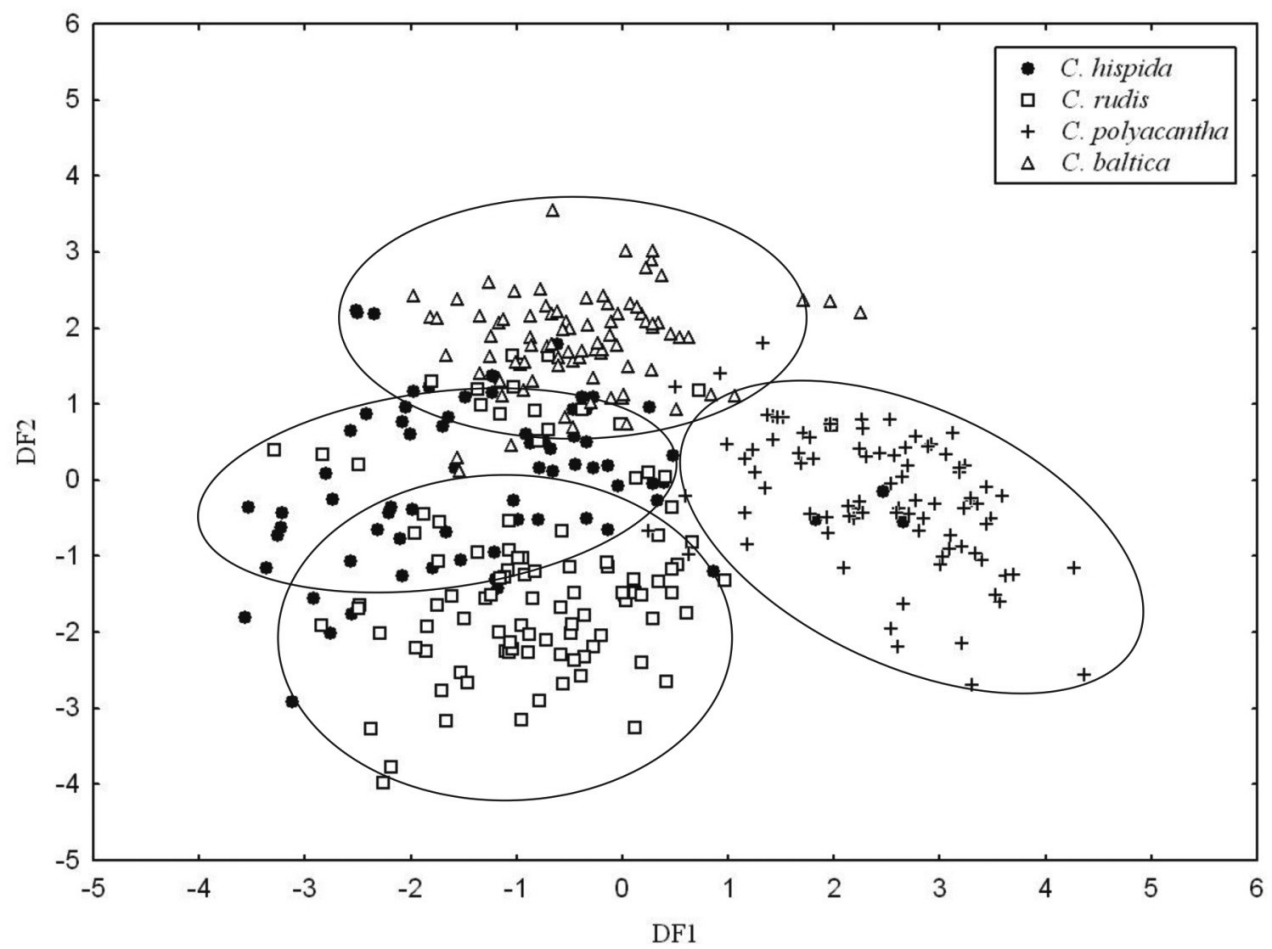
Discriminant analysis of the species from section Hartmania.

In the discriminant analysis, specimens were assigned to species groups on the basis of the classical taxonomic approach. After analysis, the first three canonical functions accounted for 96% of the total variation (first 46%, second 24% and third 24%). The analysis showed that 11 out of the 14 characters were useful for differentiating the specimens. The other characters were not significant. The individuals of *C. polyacantha*, *C. baltica* and *C. rudis* form well-separated groups, and *C. hispida* overlaps *C. rudis* and *C. baltica* (Fig. 10).

**Figure 11.**
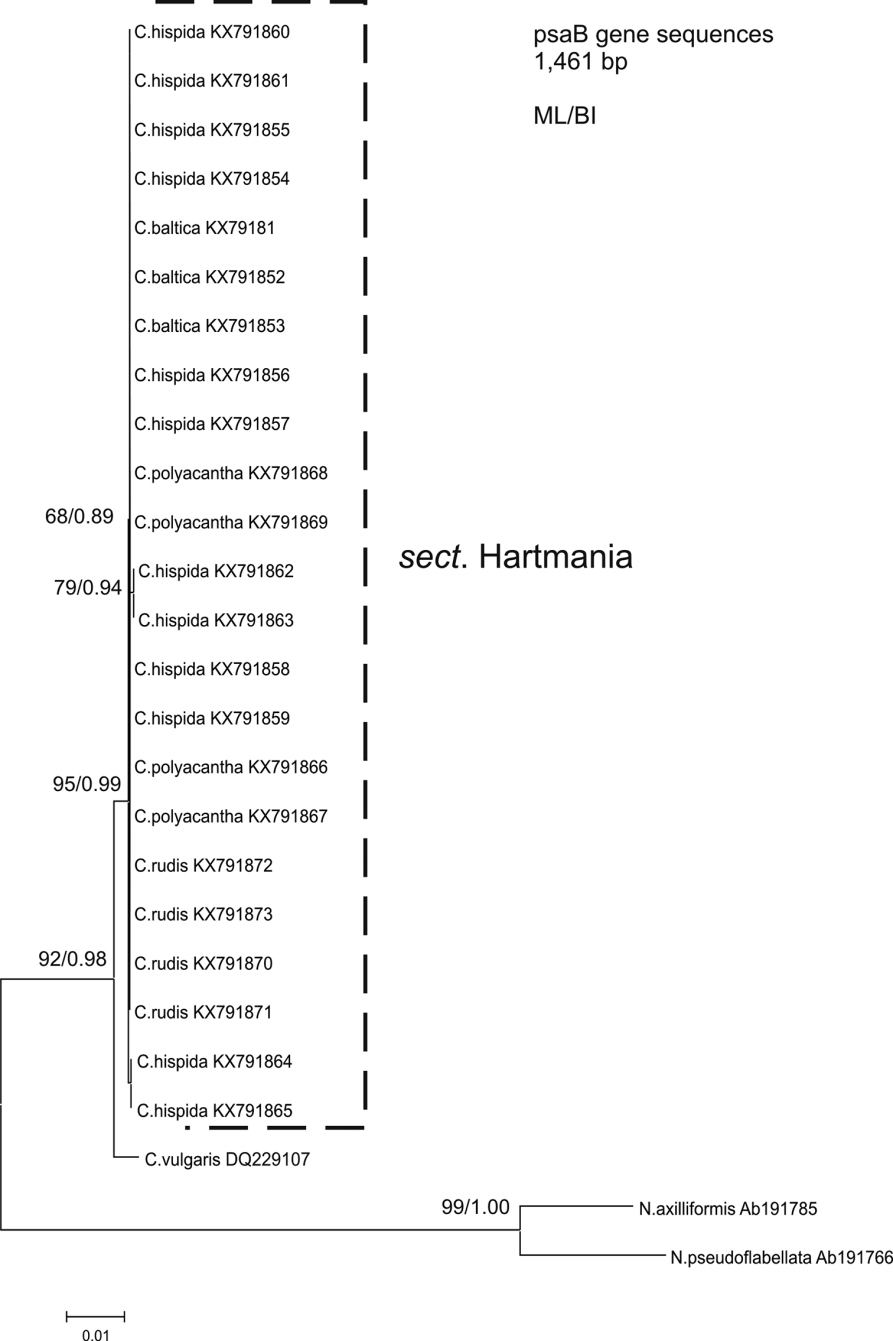
Phylogenetic tree inferred from maximum-likelihood (ML) analysis of *psa*B gene sequence data for the Charophyceae (Characeae) and outgroup taxa, with LM bootstrap support (BP)/bayesian interference (BI) indicated at the nodes.

Analysis of the *psa*B gene of *Chara* species showed a smaller resolution than on the tree produced with sequences from the *Nitella* genus (Sakayama et al. 2009). Out of the 1,461 analysed base pairs included in the *psa*B sequence analyses, 157 were informative with respect to parsimony. Almost all investigated specimens formed one congruent and unresolved clade that group all of the studied specimens. A phylogenetical tree based on the *psa*B sequences is shown in Fig. 11, and as can be seen, the four studied species belonging to section Hartmania (*C. baltica*, *C. hispida*, *C. polyacantha* and *C. rudis*) form a coherent group with high bootstrap support in ML and BI analyses. The *psa*B sequences were almost identical with no nucleotide differentiation between species.

## Discussion

The results of numerous studies indicate that a combination of various morphological data with molecular sequences can be helpful for distinguishing charophyte species, as well as making various taxonomic decisions or explaining the phylogenetic relationship between species (Sakayama et al. 2005; Urbaniak 2010; Urbaniak and Blazencic 2012).

In comparison to other authors, and especially to more recently published data on the morphological features of charophytes (Becker et al. 2016), we have observed several differences in plant characters. The specimens of *C. baltica* presently growing in the Polish part of the Baltic Sea are in general of similar length as presented in Becker et al. (2016) and no plants that reach 90 cm (i.e. C. baltica var. liliebladi) have been observed. In the case of *C. hispida* and *C. rudis*, oogonia, antheridia and oospores are in general of similar size, except for the length of oogonia measured in Polish *C. hispida* that can be shorter (minimum size 415 μm) than described in Becker et al. (2016). These authors described oogonia of *C. rudis* with a minimum breadth of 600 μm (Becker et al. 2016) whereas the Polish specimens were smaller (minimum breadth 415 µm). Both examples show how big the differences can be in measurements of oospores, oogonia and antheridia in charophytes.

The multivariate analysis of *C. hispida* and *C. rudis* based on vegetative traits gives some additional explanation of the taxonomy of species belonging to section Hartmania. The result obtained during the DCA seems to be clearer than obtained from the PCA, probably due to the different algorithms used in the analyses that can explain the differences: DCA emphasises characters that distinguish groups while suppressing the variation within groups, whereas PCA tends to accentuate the within-group variation (Řepka 2003). In general, analyses of the morphological characters show that the studied species form more or less separated groups, but all seem to be closely related.

The PCA results were used to demonstrate the differences among the species, and most specimens could be allocated to particular taxa. Both figures support the amalgamation of *C. hispida* and *C. rudis*, and maintenance of the species *C. baltica* and *C. polyacantha* as similar to how they were presented previously (Urbaniak 2010). On the other hand, several specimens were mis-allocated along the first and third axes (Fig. 9). DCA demonstrated close relations among taxa, but material assigned to *C. polyacantha* formed the most separated group. The other three species groups: *C. baltica*, *C. hispida* and *C. rudis* formed closely related groups. Despite that, *C. baltica* can reliably be distinguished by a combination of morphological characters and by their occurrence in different habitats (Krause 1997; Urbaniak and Gąbka 2014). *C. baltica* and *C. polyacantha* differ greatly in their morphology and both taxa are differentiated not only by morphological characters but also by ecological preferences. *C. baltica* is a typically brackish water species, whereas *C. polyacantha*, *C. hispida* and *C. rudis* are typically fresh water species. This contrasts with Wood and Imahori (1965) who treated these taxa as varieties of *C. hispida* (Table 2). However, despite good segregation of the majority of specimens in these two species, there are still a number of specimens that have overlapping characteristics. This is likely to be a result of a close phylogenetic relationship between those species (Boegle et al. 2007), which has also been contradicted by the present results.

On the other hand, it supports the thesis that all these species are morphologically very similar, and that ‘transitional forms’ commonly exist between them. The so-called ‘transitional forms’ are probably not real hybrids, but rather forms that visualize possible plasticity that can be noted in the genus *Chara*. In this group of species: *C. hispida*, *C. rudis*, *C. polyacantha* and *C. baltica* ‘transitional forms’ are those that display features intermediate between species, or the features are not clear enough for determination. In the case of *C. rudis* and *C. hispida*, spine cells are the main distinguishing character, and they are normally in twos or threes in *C. hispida*, but in pairs lying one above the other along the axis in *C. rudis* (Urbaniak and Gąbka 2014), Fig. 7. Both features can be found on the same plant, and this sometimes makes determination difficult or impossible. The transitional forms of *Chara* species do not grow only as morphologically mixed populations. They can occur also in populations where most of the specimens are easily allocated to one species or the other. Quantitative characters, including those of cortication and general appearance of habit, are generally very variable and cannot offer reliable characters for determination. Earlier authors did not always use all the characters identified as important by DCA in this work. This could be quite complicated in the routine determination but may be necessary to reach a proper understanding of the taxonomy of the group (Urbaniak 2010); however, a really deep understanding of the taxonomic relationships among the group depends on both molecular and morphological studies on different populations of species within the section Hartmania.

The analysis of phylogenetic sequence data reveals a strictly close relationship between *C. baltica*, *C. hispida*, *C. polyacantha* and *C. rudis* (Fig. 11). The results based on the *psa*B cpDNA sequences show one clade on the phylogenetic tree, which is not exactly congruent with the morphological analyses, but contradicts the previously found taxonomic relations between species (Urbaniak 2011a, 2011b; Urbaniak and Combik 2013). Our *psa*B phylogeny clearly revealed that the species of section Hartmania are monophyletic and the groups of sequences (section Hartmania) form a cluster containing all individuals together. The lack of genetic variability in them did not differ at all in the species and showed a lack of discrimination, as similar as in Schneider et al. (2015), who found that one large and unresolved group consisted of species such as *C. intermedia*, *C. hispida*, *C. horrida*, *C. baltica*, *C. polyacantha* and *C. rudis*. Results based on more data analysed showed that many more species that can differ morphologically or genetically are placed in *C. hispida* cluster (Schneider et al. 2016). This, in particular, can contradict that all studied species are very closely related, but on the other hand, the *psaB* seems to be not the best marker for studying phylogenetic and taxonomic relations between species from the genus *Chara*. This, however, is not in accordance with the previous work e.g. on the genera *Nitella* (charophyta). Sakayama et al. (2005) found that *psa*B can concatenate with other genes or morphological analysis of oospore wall ornamentation gave successful discrimination, but in presented results, morphologically different species were not differentiated by molecular analysis. This could rather support the hypothesis on the close phylogenetical and evolutionary relations that exist between species from the section Hartmania.

Although morphological and molecular data separately are not ideal tools for species delimitation, together they are important and useful when combined with other types of data (Sakayama 2008). Such studies are being published at an increasing rate and are discovering cryptic species (Bickford et al. 2007). Lack of differentiation based on barcodes or fingerprinting techniques allows for the reinterpretation of some particular taxa in the charophytes, particularly in the genus *Chara*. The obtained results show that close taxonomic relations between studied species are not questionable, however, more adequate data, used molecular markers and performed on a wider spectrum of taxa, are needed for a better understanding of such relations.

## Conclusion

We have shown that morphological features allow for differentiation of the investigated *Chara* species. *C. polyacantha* formed separate clusters in both PCA and DCA, and *C. rudis* had intermediate features. Molecular analyses showed that all species definitely comprise one closely related group and no differentiation in the *psa*B variability between them has been found.
